# Modulation of Glutamate Transporter EAAT1 and Inward-Rectifier Potassium Channel K_ir4.1_ Expression in Cultured Spinal Cord Astrocytes by Platinum-Based Chemotherapeutics

**DOI:** 10.3390/ijms22126300

**Published:** 2021-06-11

**Authors:** Markus Leo, Linda-Isabell Schmitt, Rebecca Steffen, Andrea Kutritz, Christoph Kleinschnitz, Tim Hagenacker

**Affiliations:** Department of Neurology, Center for Translational Neuro- and Behavioral Sciences (C-TNBS), University Hospital Essen, 45147 Essen, Germany; Linda-Isabell.Schmitt@UK-Essen.de (L.-I.S.); Rebecca.Steffen@stud.uni-due.de (R.S.); Andrea.Kutritz@UK-Essen.de (A.K.); Christoph.Kleinschnitz@UK-Essen.de (C.K.); Tim.Hagenacker@UK-Essen.de (T.H.)

**Keywords:** chemotherapy-induced neuropathy, spinal cord, astrocytes, cisplatin, oxaliplatin, potassium channel, GFAP, EAAT1, K_ir4.1_

## Abstract

Platinum-based chemotherapeutics still play an essential role in cancer treatment. Despite their high effectiveness, severe side effects such as chemotherapy-induced neuropathy (CIPN) occur frequently. The pathophysiology of CIPN by platinum-based chemotherapeutics is not fully understood yet, but primarily the disturbance of dorsal root ganglion cells is discussed. However, there is increasing evidence of central nervous system involvement with activation of spinal cord astrocytes after treatment with chemotherapeutics. We investigated the influence of cis- or oxaliplatin on the functionality of cultured rat spinal cord astrocytes by using immunocytochemistry and patch-clamp electrophysiology. Cis- or oxaliplatin activated spinal astrocytes and led to downregulation of the excitatory amino acid transporter 1 (EAAT1) expression. Furthermore, the expression and function of potassium channel K_ir4.1_ were modulated. Pre-exposure to a specific Kir4.1 blocker in control astrocytes led to a reduced immune reactivity (IR) of EAAT1 and a nearly complete block of the current density. When spinal astrocytes were pre-exposed to antibiotic minocycline, all effects of cis- or oxaliplatin were abolished. Taken together, the modulation of K_ir4.1_ and EAAT1 proteins in astrocytes could be linked to the direct impact of cis- or oxaliplatin, identifying spinal astrocytes as a potential target in the prevention and treatment of chemotherapy-induced neuropathy.

## 1. Introduction

Platinum-based chemotherapeutics as cis- or oxaliplatin still play an essential role in treating different cancer types today. Besides its efficacy in tumor treatment, cis- or oxaliplatin leads to severe neurotoxic side effects, resulting in chemotherapy-induced neuropathy (CIPN) [1,2,3]. CIPN’s typical symptoms are impaired sensory function, manifested in numbness, tingling, spontaneous feeling of touch, and paresthesia [4,5]. Furthermore, painful sensations such as non-evoked burning, shooting, and mechanical or thermal allodynia or hyperalgesia occur frequently [1,4,6,7,8,9]. The drug-specific pathomechanisms leading to CIPN are not fully understood, but several are suggested, such as calcium disturbance in dorsal root ganglia neurons, mitochondrial dysfunctions, or also activation of satellite glial cells [10,11,12,13,14,15]. However, treatment with paclitaxel and oxaliplatin also shows the involvement of the central nervous system (CNS) in the activation of spinal cord astrocytes [16,17].

Astrocytes play an essential role in the CNS, expressing various types of neuro- or gliotransmitters, pro-inflammatory cytokines, and growth factors [18,19,20,21]. Furthermore, astrocytes are part of the blood-brain-barrier and are involved in water homeostasis. Astrocytes are crucial for spatial potassium buffering, which is essential for controlling extracellular potassium concentration and neuronal excitability [22,23]. Spatial potassium buffering in astrocytes is mainly mediated by inwardly rectifying potassium channel K_ir4.1_ [22]. This channel has been shown to be modulated in satellite glial cells (SGCs) of the peripheral nervous system (PNS), a glial cell type with similar functions to the astrocytes of the CNS, in different pain conditions as CIPN [11,24,25,26].

Additionally, the spatial potassium buffer system is coupled to astrocytic glutamate uptake from the extracellular room by glutamate transporters, such as the excitatory amino acid transporter 1 (EAAT1). Disturbances of the EAAT1 function can result in a toxic increase of extracellular glutamate concentration, leading to enhanced neuronal activity and excitatory toxicity [27,28,29,30,31].

In CIPN, it is unclear if spinal astrocytes are activated by substances released from the central synaptic endings of peripheral sensory neurons or directly by chemotherapeutics. For a long time, it was suggested that chemotherapeutics such as cisplatin, oxaliplatin, or paclitaxel were not capable of entering the CNS, but in recent studies, small amounts of chemotherapeutics were detected in the brain and spinal cord [32,33,34].

This study investigated the direct influence of cis- or oxaliplatin concentrations on isolated spinal astrocytes. Here we focused on astrocyte-activation, the expression of EAAT1 and K_ir4.1_, and its electrophysiological function. Therefore, we used immunocytochemical staining and patch-clamp electrophysiology. Furthermore, we evaluated a direct relationship between the modulation of K_ir4.1_ and EAAT1 expression using a selective K_ir4.1_ channel inhibitor.

## 2. Results

### 2.1. Isolation of Spinal Astrocytes from Juvenile Rats

**Figure 1:**
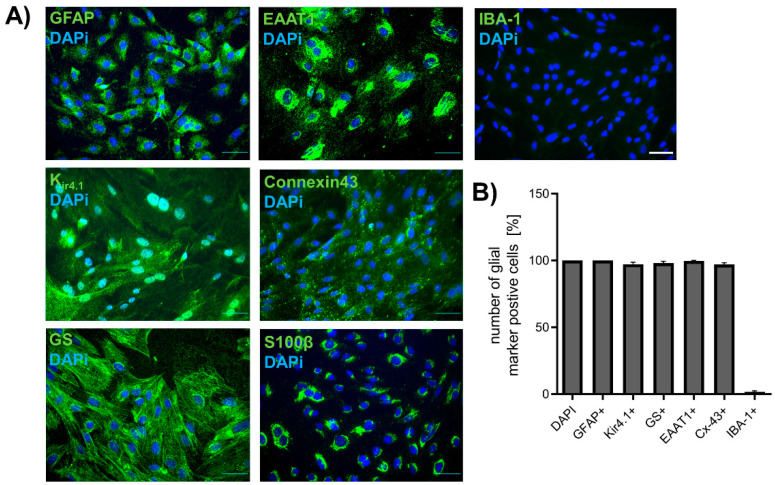
Isolation of spinal astrocytes from rats. (**A**) Immunocytochemical staining of different markers for astrocytes (GFAP, K_ir4.1_, GS, EAAT1, Cx-43) and microglia (IBA-1) cultured cells from spinal cords of rats. (**B**) The percentage of GFAP-, K_ir4.1_-, GS-, EAAT1-, or Cx43-positive cells were >97%, while only <2% of the cells were positive for IBA1. *n* = 4 individual experiments for each marker with >100 cells. Scale 50 µm.

The purity of spinal astrocyte cultures was validated by immunocytochemical staining (ICC) for different glial-markers such as GFAP, GS, EAAT1, K_ir4.1_, Cx-43, and S100β. To evaluate the number of contaminating microglia, staining for IBA-1 was performed (Figure 1A). Immunocytochemical staining confirmed the successful isolation and cultivation of astrocytes from rat spinal cords. Isolated cells were positive for GFAP (100.00 ± 0.0%), K_ir4.1_ (97.15 ± 1.49%), GS (98.04 ± 1.42%), EAAT1 (99.69 ± 0.31%), Cx-43 (97.09 ± 1.18%), and S100β (100.00 ± 0.00%). Only 1.63 ± 0.75% of the cells were positive for microglia marker IBA-1 (Figure 1B). No increase of Iba-1 positive cells in astrocyte cultures was observed after exposure to cis- or oxaliplatin for 24 h (Figure S1, Supplementary Material).

### 2.2. Activation of Spinal Astrocytes after Exposure to Chemotherapeutics

**Figure 2:**
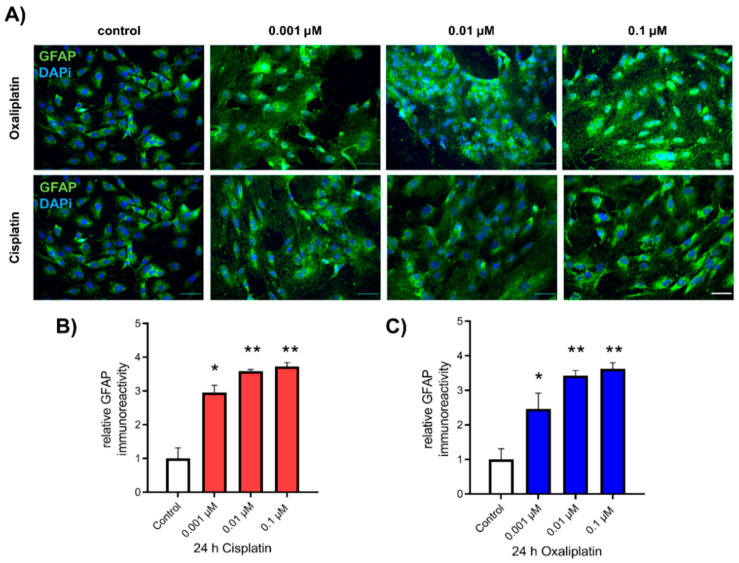
Expression of GFAP in cultures of spinal Astrocytes before and after exposure to cis- or oxaliplatin. (**A**) Immunocytochemical staining of GFAP (green) in cultured spinal astrocytes before and after exposure to 0.001 µM, 0.01 µM, or 0.1 µM cis- or oxaliplatin for 24 h. Nuclear DNA was stained with Dapi (blue). (**B**,**C**) Exposure to all concentrations of cis- or oxaliplatin leads to an increase of the GFAP expression in spinal astrocytes after 24 h (* *p* < 0.05; ** *p* < 0.01). * = significant difference to control. *n* = 9 individual experiments with >100 cells each. Scale 50 µm.

For examining the influence of cis- or oxaliplatin on the expression of GFAP in spinal astrocytes, as a marker for activation, cells were cultured and exposed to cis- or oxaliplatin in a concentration of 0.001 µM, 0.01 µM, or 0.1 µM for 24 h. Afterward, ICC for GFAP immunoreactivity was performed (Figure 2A). Untreated spinal astrocytes showed a GFAP protein level of 1.00 ± 0.31. When spinal astrocytes were exposed to cisplatin, all concentrations led to an increased relative GFAP protein level (0.001 µM: 2.95 ± 0.22, *p* < 0.05; 0.01 µM: 3.59 ± 0.05, *p* < 0.01; 0.1 µM: 3.72 ± 0.12, *p* < 0.01) (Figure 2B). Exposure of spinal astrocytes to oxaliplatin for 24 h showed similar results. The relative GFAP protein level was increased (0.001 µM: 2.46 ± 0.25, *p* < 0.05; 0.01 µM: 3.42 ± 0.15, *p* < 0.01; 0.1 µM: 3.62 ± 0.17, *p* < 0.01), compared to the control condition (Figure 2C). Additionally, expression of S100β and the production of reactive oxygen species (ROS), as other markers of astrocyte activity, were increased by cis- or oxaliplatin after 24 h (Figures S2 and S3, Supplementary Material).

### 2.3. Reduction of K_ir4.1_ Protein Expression after Exposure to Chemotherapeutics

**Figure 3:**
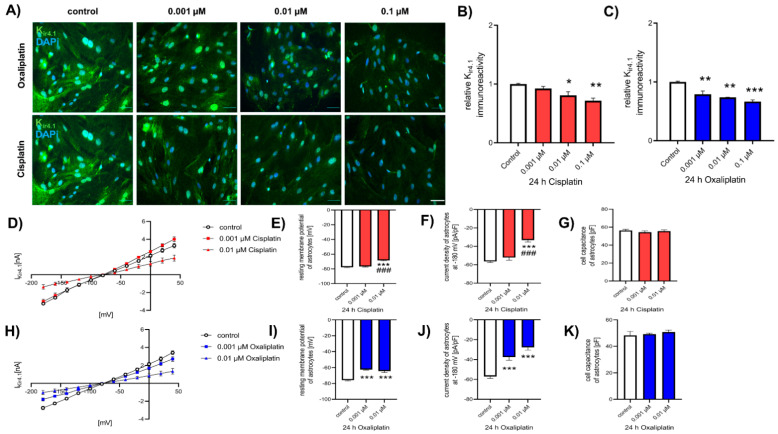
Expression and function of K_ir4.1_ in cultures spinal astrocytes before and after exposure to cis- or oxaliplatin. (**A**) Immunocytochemical staining of K_ir4.1_ (green) in cultured spinal astrocytes before and after exposure to 0.001 µM, 0.01 µM, or 0.1 µM cis- or oxaliplatin for 24 h. Nuclear DNA was stained with Dapi (blue). (**B**) Exposure to 0.01 µM (* *p* < 0.05) or 0.1 µM (** *p* < 0.01) cisplatin led to a reduction of the K_ir4.1_ expression after 24 h, while 0.001 µM cisplatin did not show an effect. (**C**) Exposure to all concentrations of oxaliplatin reduced the K_ir4.1_ expression after 24 h (** *p* < 0.01; *** *p* < 0.001), compared to untreated control cells. *n* = 9 individual experiments with >100 cells each. Scale 50 µm. (**D**) Current-voltage relation curve of K_ir4.1_ in spinal astrocytes under control conditions or after exposure to 0.001 µM or 0.01 µM cisplatin for 24 h. (**E**) Exposure to 0.01 µM cisplatin for 24 h reduced the resting membrane potential of spinal astrocytes (*** *p* < 0.001; ### *p* < 0.001), compared to 0.001 µM cisplatin (*p* > 0.05) or untreated control cells. (**F**) The current density of K_ir4.1_ was not affected when spinal astrocytes were exposed to 0.001 µM cisplatin for 24 h (*p* > 0.05), compared to control conditions. When cells were exposed to 0.01 µM cisplatin, the current density was reduced (*** *p* < 0.001; ### *p* < 0.001). (**G**) Cell capacity of spinal astrocytes was not affected by cisplatin (*p* > 0.05). (**H**) Current-voltage relation curve of K_ir4.1_ in spinal astrocytes under control conditions or after exposure to 0.001 µM or 0.01 µM oxaliplatin for 24 h. (**I**) Exposure to 0.001 µM or 0.01 µM oxaliplatin for 24 h reduced the resting membrane potential of spinal astrocytes (*** *p* < 0.001), compared to untreated control cells. (**J**) The current density of K_ir4.1_ was reduced when spinal astrocytes were exposed to 0.001 µM or 0.01 µM oxaliplatin for 24 h (*** *p* < 0.001), compared to control conditions. (**K**) Cell capacity of spinal astrocytes was not affected by oxaliplatin (*p* > 0.05). * = significant difference to control. # = significant effect to previous concentration. *n* = 6 cells each condition.

To investigate the effect of cis- or oxaliplatin on the protein level of inward-rectifier potassium channel K_ir4.1_, spinal astrocytes were cultured and exposed to chemotherapeutic drugs, as described before, and ICC was performed (Figure 3A). Under control conditions, the K_ir4.1_ protein level was 1.00 ± 0.01. Exposure of spinal astrocytes to 0.01 µM or 0.1 µM cisplatin for 24 h resulted in a reduction of relative K_ir4.1_ protein level (0.01 µM: 0.81 ± 0.06, *p* < 0.05; 0.1 µM: 0.72 ± 0.04, *p* < 0.01). In contrast, a concentration of 0.001 µM cisplatin did not affect the K_ir4.1_ protein level (0.92 ± 0.03, *p* > 0.05), compared to the control condition (Figure 3B). When cells were exposed to oxaliplatin, a reduction of relative K_ir4.1_ protein level was observed for all used concentrations (0.001 µM: 0.92 ± 0.03, *p* < 0.01; 0.01 µM: 0.81 ± 0.06, *p* < 0.01; 0.1 µM: 0.72 ± 0.04, *p* < 0.001) (Figure 3C).

To evaluate the function of K_ir4.1_, patch-clamp measurements were performed (Figure 3D,H). When cultured spinal astrocytes were exposed to 0.01 µM cisplatin, the resting membrane potential was reduced (−68.36 ± 0.67 mV; *p* < 0.001), compared to the control conditions (−77.71 ± 0.66 mV) and to cells exposed to 0.001 µM cisplatin (−76.70 ± 0.70 mV, *p* > 0.05) (Figure 3E). Similar effects were observed for the current density; 0.001 µM cisplatin did not lead to an effect (−54.20 ± 2.89 pA/pF, *p* > 0.05), while 0.01 µM cisplatin reduced the current density of K_ir4.1_ (−33.24 ± 2.09 pA/pF, *p* < 0.001), compared to untreated control cells (−56.35 ± 1.29 pA/pF) (Figure 3F). Exposure of spinal astrocytes to cisplatin did not alter the capacity of the cells after 24 h (*p* > 0.05) (Figure 3G). When cultured astrocytes were exposed to oxaliplatin, all concentrations reduced the resting membrane potential (0.001 µM: −62.48 ± 0.67 mV, *p* < 0.001; 0.01 µM: −64.42 ± 1.72 mV; *p* < 0.001), compared to untreated cells (−78.88 ± 0.91 mV) (Figure 3I). The current density of K_ir4.1_ in spinal astrocytes was also reduced by exposure to 0.001 µM (−37.46 ± 3.08 pA/pF, *p* < 0.001) or 0.01 µM (−27.84 ± 2.75 pA/pF, *p* < 0.001) oxaliplatin (Figure 3J). Similar to cisplatin, the cell capacity of spinal astrocytes was not altered by oxaliplatin (*p* > 0.05) (Figure 3K).

### 2.4. Reduction of EAAT1 Protein Expression after Exposure to Chemotherapeutics

**Figure 4:**
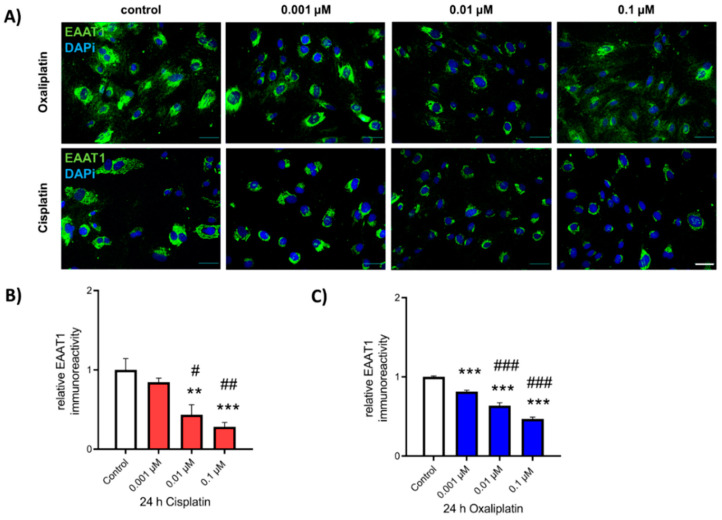
Expression of EAAT1 in cultured spinal astrocytes before and after exposure to cis- or oxaliplatin. (**A**) Immunocytochemical staining of EAAT1 (green) in cultured spinal astrocytes before and after exposure to 0.001 µM, 0.01 µM, or 0.1 µM cis- or oxaliplatin for 24 h. Nuclear DNA was stained with Dapi (blue). (**B**) Exposure of spinal astrocytes to 0.001 µM cisplatin did not affect the expression of EAAT1 (*p* > 0.05). When cells were exposed to 0.01 µM (** *p* < 0.01; # *p* < 0.05) or 0.1 µM (*** *p* < 0.001; ## *p* < 0.01) cisplatin, EAAT1 expression was reduced. (**C**) Exposure of spinal astrocytes to all concentrations of oxaliplatin for 24 h led to a reduction of EAAT1 expression (*** *p* < 0.001; ### *p* < 0.001). * = significant difference to control. # = significant difference to previous concentration. *n* = 9 individual experiments with >100 cells. Scale 50 µm.

To determine the possible effects of cis- or oxaliplatin on the expression of glutamate-transporter EAAT1, cultured spinal astrocytes were exposed to chemotherapeutics as described above, and ICC for EAAT1 immunoreactivity was performed (Figure 4A). When spinal astrocytes were exposed to 0.01 µM or 0.1 µM of cisplatin for 24 h, relative EAAT1 protein level was reduced (0.01 µM: 0.43 ± 0.13, *p* < 0.01; 0.1 µM: 0.28 ± 0.05, *p* < 0.001), compared to untreated conditions (1.00 ± 0.01). No effect was observed when cells were exposed to 0.001 µM cisplatin (0.85 ± 0.05, *p* > 0.05). A concentration-dependent effect on the reduction of the EAAT1 IR was observed between 0.01 µM and 0.1 µM cisplatin (*p* < 0.05—*p* < 0.01) (Figure 4B). In contrast to cisplatin, exposure to oxaliplatin resulted in a concentration-dependent reduction of relative EAAT1 protein level for all concentrations (0.001 µM: 0.82 ± 0.02, *p* < 0.001; 0.01 µM: 0.64 ± 0.04, *p* < 0.001; 0.1 µM: 0.47 ± 0.03, *p* < 0.001) (Figure 4C).

### 2.5. Reduction in EAAT1 Protein Expression Is Mediated by K_ir4.1_ Dysfunction

**Figure 5:**
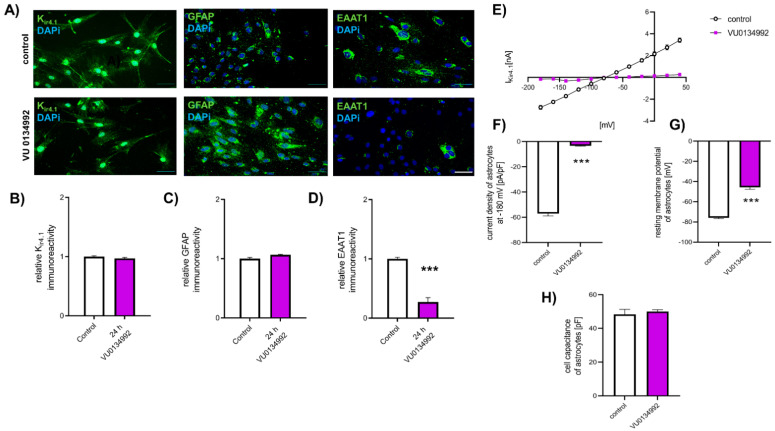
Influence of VU0134992 on the expression and function of K_ir4.1_, GFAP, or EAAT1 in cultured spinal astrocytes. (**A**) Immunocytochemical staining of K_ir4.1_ (green) in cultured spinal astrocytes with or without exposure to K_ir4.1_ blocker VU0134992 for 24 h. Nuclear DNA was stained with Dapi (blue). (**B**) Exposure to VU0134992 for 24 h did not affect the expression of K_ir4.1_ in cultured spinal astrocytes (*p* > 0.05). (**C**) GFAP expression was not affected by VU0134992 exposure for 24 h (*p* > 0.05). (**D**) Exposure to VU0134992 for 24 h led to a reduction of EAAT1 expression (*** *p* < 0.001. *n* = 9 experiments with each >100 cells. Scale 50 µm. (**E**) Current-voltage relation curve of K_ir4.1_ in spinal astrocytes under control conditions or after exposure to VU0134992 for 24 h. (**F**) Exposure to VU0134992 reduced current density of K_ir4.1_ in spinal astrocytes after 24 h (*** *p* < 0.001). (**G**) Blockade of K_ir4.1_ by VU0134992 led to reduced spinal astrocytes’ resting membrane potential after 24 h (*** *p* < 0.001). (**H**) The cell capacity of spinal astrocytes was not affected by exposure to VU0134992 (*p* > 0.05). * = significant difference to control. *n* = 6 cells each condition.

To examine the effect of K_ir4.1_ function on EAAT1 expression and for showing a relation between those two proteins, cultured spinal astrocytes were exposed to K_ir4.1_ specific inhibitor VU01344992 (VU) for 24 h. Afterward, the relative K_ir4.1_ and EAAT1 protein levels were evaluated by ICC (Figure 5A). Here, no effect of blocking K_ir4.1_ function by VU on K_ir4.1_ protein level was observed (0.92 ± 0.01, *p* > 0.05), compared to untreated conditions (1.00 ± 0.02) (Figure 5B). Furthermore, no effect of VU on the GFAP protein level was observed (*p* > 0.05) (Figure 5C). In contrast, the relative protein level of EAAT1 in spinal astrocytes was reduced 24 h after exposure to VU (VU: 0.27 ± 0.07; control: 1.00 ± 0.27, *p* < 0.001) (Figure 5D).

The influence of VU on the function of K_ir4.1_ was examined by patch-clamp measurements (Figure 5E). Inhibition of K_ir4.1_ by VU led to a reduction of the current density (−3.31 ± 0.41 pA/pF, *p* < 0.001), compared to untreated cells (−57.01 ± 1.92 pA/pF) (Figure 5F). Additionally, the resting membrane potential of spinal astrocytes was reduced by exposure to VU after 24 h (Ctl: −75.88 ± 0.91 mV; VU: −45.62 ± 2.1 mV, *p* < 0.001) (Figure 5G). In contrast, the cell capacity of spinal astrocytes was not affected by VU (*p* > 0.05) (Figure 5H).

### 2.6. Chemotherapeutic Effects on Spinal Astrocytes Can Be Prevented by Antibiotic Minocycline

To investigate the potential of minocycline as a protective agent, spinal astrocytes were cultured as described above and pre-exposed to 50 µg/mL minocycline 20 min before cells were exposed to cis- or oxaliplatin for 24 h. Afterward, ICC against GFAP, K_ir4.1_, and EAAT1 was performed. Furthermore, K_ir4.1_ current density was measured. Minocycline did not affect the function of control astrocytes (*p* > 0.05).

**Figure 6:**
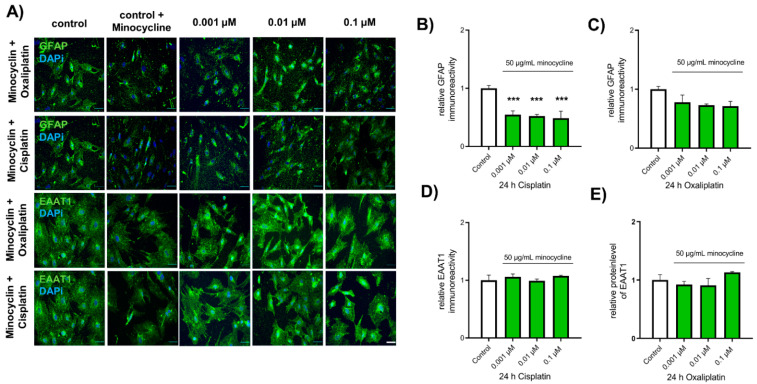
Influence of antibiotic minocycline on the expression of GFAP and EAAT1 in cultured spinal astrocytes. (**A**) Immunocytochemical staining of GFAP or EAAT1 (green) in cultured spinal astrocytes before or after exposure to minocycline and cis- or oxaliplatin. Nuclear DNA was stained with Dapi (blue). (**B**) When spinal astrocytes were pre-exposed to minocycline, cisplatin reduced the expression of GFAP compared to untreated control cells (*** *p* < 0.001). (**C**) When spinal astrocytes were pre-exposed to minocycline, oxaliplatin did not affect the expression of GFAP compared to untreated control cells (*p* > 0.05). (**D**,**E**) Cis- or oxaliplatin did not affect the expression of EAAT1 when spinal astrocytes were pre-exposed to minocycline (*p* > 0.05). * = significant difference to control. *n* = 9 experiments with each >100 cells. Scale 50 µm.

When spinal astrocytes were pre-treated with minocycline, exposure to cisplatin led to a reduction of GFAP expression, compared to untreated control cells (Ctl: 1 ± 0.05; 0.001 µM: 0.55 ± 0.07, *p* < 0.001; 0.01 µM: 0.52 ± 0.03, *p* < 0.001; 0.1 µM: 0.49 ± 0.12, *p* < 0.001). When minocycline-treated spinal astrocytes were exposed to oxaliplatin, no alteration in GFAP expression was observed, compared to control cells (Ctl: 1 ± 0.05; 0.001 µM: 0.78 ± 0.12, *p* > 0.05; 0.01 µM: 0.73 ± 0.02, *p* > 0.05; 0.1 µM: 0.71 ± 0.06, *p* > 0.05) (Figure 6A–C).

Furthermore, minocycline pre-treatment protects spinal astrocytes from chemotherapeutic-mediated downregulation of EAAT1 expression. No effect on EAAT1 expression was observed when minocycline pre-treated spinal astrocytes were exposed to cisplatin (0.001 µM: 1.06 ± 0.05, *p* > 0.05; 0.01 µM: 0.99 ± 0.03, *p* > 0.05; 0.1 µM: 1.08 ± 0.10, *p* > 0.05) or oxaliplatin (0.001 µM: 0.92 ± 0.06, *p* > 0.05; 0.01 µM: 0.91 ± 0.12, *p* > 0.05; 0.1 µM: 1.13 +0.01, *p* > 0.05), compared to untreated cells (1 ± 0.09) (Figure 6A,D,E).

**Figure 7:**
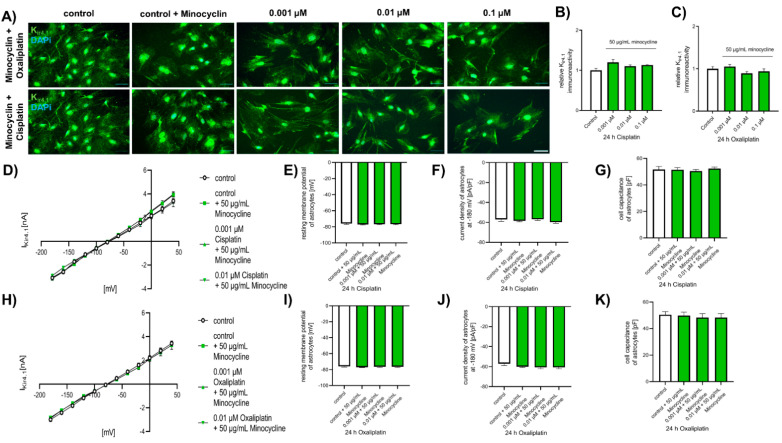
Influence of antibiotic minocycline on the expression and function of K_ir4.1_ in cultured spinal astrocytes. (**A**) Immunocytochemical staining of K_ir4.1_ (green) in cultured spinal astrocytes before or after exposure to minocycline and cis- or oxaliplatin. Nuclear DNA was stained with Dapi (blue). (**B**,**C**) Cis- or oxaliplatin did not affect the expression of K_ir4.1_ when spinal astrocytes were pre-exposed to minocycline (*p* > 0.05). *n* = 9 experiments with each >100 cells. Scale 50 µm. (**D**) Current-voltage relation curve of K_ir4.1_ in spinal astrocytes under control conditions or after exposure to minocycline and 0.001 µM or 0.1 µM cisplatin for 24 h. (**E**–**G**) When spinal astrocytes were pre-exposed to minocycline, cisplatin did not affect the resting membrane potential (*p* > 0.05), the current density of K_ir4.1_ (*p* > 0.05), and the cell capacity (*p* > 0.05). (**H**) Current-voltage relation curve of K_ir4.1_ in spinal astrocytes under control conditions or after exposure to minocycline and 0.001 µM or 0.1 µM oxaliplatin for 24 h. (**I**–**K**) When spinal astrocytes were pre-exposed to minocycline, oxaliplatin did not affect the resting membrane potential (*p* > 0.05), the current density of K_ir4.1_ (*p* > 0.05), and the cell capacity (*p* > 0.05). *n* = 6 cells each condition.

Additionally, chemotherapeutic effects on K_ir4.1_ expression were abolished by minocycline pre-exposure. When spinal astrocytes were pre-treated with minocycline, exposure to cisplatin did not lead to alteration of K_ir4.1_ protein level (Ctl: 1 ± 0.05; 0.001 µM: 1.21 ± 0.08, *p* < 0.05; 0.01 µM: 1.03 ± 0.03, *p* > 0.05; 0.1 µM: 1.13 ± 0.01, *p* > 0.05). A similar result was observed when pre-treated spinal astrocytes were exposed to oxaliplatin (Ctl: 1 ± 0.05; 0.001 µM: 1.06 ± 0.05, *p* > 0.05; 0.01 µM: 0.89 ± 0.04, *p* > 0.05; 0.1 µM: 0.94 ± 0.06, *p* > 0.05) (Figure 7A–C).

To examine the protective influence of minocycline on the functional modulation of K_ir4.1_, mediated by chemotherapeutics, patch-clamp measurements were performed. All observed effects of cis- or oxaliplatin on the resting membrane potential (*p* > 0.05) and the current density of K_ir4.1_ (*p* > 0.05) were abolished by pre-exposure to minocycline. Furthermore, the cell capacity was not affected by cis- or oxaliplatin (Figure 7D–K).

## 3. Discussion

The contribution of the CNS to different pain syndromes is well known. The entrance of chemotherapeutic drugs such as cis- or oxaliplatin into the CNS is still a controversial topic that is often discussed. Recent studies have provided evidence for those drugs’ capability to reach the CNS by small concentrations of unknown biological significance. Our study demonstrates a direct influence of the chemotherapeutic drugs cis- and oxaliplatin on the expression and function of proteins such as GFAP, K_ir4.1_, and EAAT1 in spinal astrocytes, suggesting a potential role for those cells in CIPN. We describe a direct relation between glutamate transporter EAAT1 expression and the function of potassium channel K_ir4.1_, independent of GFAP upregulation. Furthermore, we show protection of spinal astrocyte functions by minocycline after exposure to chemotherapeutic drugs.

Regarding its single cell type in vitro approach, our study does not reflect the complex situation of an in vivo model of CIPN where astrocyte’s function can be influenced by dorsal horn neurons, microglia, or cells of the peripheral nervous system. Nevertheless, with this spinal astrocyte-based in vitro approach, the direct influence on spinal astrocytes by low concentrations of platinum-based chemotherapeutics can be investigated.

Exposure to cis- or oxaliplatin resulted in increased GFAP IR in cultured spinal astrocytes, suggesting strong activation of these cells after chemotherapeutic treatment. GFAP is one of the major cytoskeletal proteins in astrocytes and is often regulated during different diseases and is linked to cell migration, the anchoring of membrane proteins as glutamate transporters or ion channels, and mitosis [35,36,37]. Different studies have shown an increase in GFAP in spinal astrocytes after in vivo oxaliplatin administration [38,39,40,41]. Yoon et al. described an activation and morphological alteration of astrocytes with thickened and elongated processes until day (d) 7 after administration, but the effects were diminished after d 14 [41]. A single injection of oxaliplatin (6 mg/kg) was demonstrated to induce mechanical and cold allodynia and increase GFAP expression in the spinal cord [42]. In vivo administration of chemotherapeutic paclitaxel to rats activated spinal astrocytes already 4 h after administration until the end of the experiment at d 28 [43]. Astrocytes are not the only glial cell type showing activation of GFAP after exposure to cis- or oxaliplatin. Cultured SGCs of the DRG also showed an upregulation of GFAP expression after cis- or oxaliplatin exposure [11,25]. As other markers of astrocyte activation, we have shown upregulation of S100β expression and ROS production. The calcium-binding protein S100β is known to be upregulated in and released from astrocytes due to injury processes [44,45,46]. An increase in ROS production has already been demonstrated in SGCs after chemotherapeutic exposure [11,25]. A relation between ROS increase and the expression of GFAP has already been described in cortical astrocytes [47]. However, a potential mechanism for astrocyte activation by chemotherapeutics has not yet been described. Different subtypes of potassium channels can be found in various cell types among the nervous system. One of these subtypes expressed in astrocytes is the potassium inward rectifier channel K_ir4.1_. Its principal function is the regulation and buffering of extracellular homeostasis [48]. We demonstrated that exposure with cis- or oxaliplatin reduces the IR of K_ir4.1_ expression in spinal astrocytes. A similar effect was reported in hyperglycaemic cultured astrocytes and in mouse models of diabetes. Hyperglycaemia downregulates K_ir4.1_ protein in vitro and in vivo, resulting in the increased spontaneous activity of surrounding neurons in the spinal cord’s dorsal horn [49]. Additionally, the reduction of K_ir4.1_ expression in SGCs has been reported in different pain models [11,24,25,50]. In SGC cell cultures, exposure with cis- or oxaliplatin in different concentrations also reduced the K_ir4.1_ IR and the protein level in WB [11,25].

In addition to the IR reduction of the K_ir4.1_, the current density and the resting membrane potentials of the spinal astrocytes were decreased after exposure with cis- or oxaliplatin for 24 h. No differences were observed in the cell capacities of spinal astrocytes when exposed to chemotherapeutics, suggesting a direct functional effect of cis- or oxaliplatin on the current density by reducing K_ir4.1_ expression and not just by a cell size mediated effect. Different pain models could show a reduction of K_ir4.1_ currents as well as altered resting membrane potentials in SGCs [11,25]. In diseases such as Alzheimer’s, Huntington’s disease, or amyotrophic lateral sclerosis (ALS), a reduction of the K_ir4.1_ expression or current density in astrocytes has been reported, resulting in hyperexcitability of the surrounding neurons [51,52,53,54,55,56].

Besides the potassium buffering, astrocytes also play a crucial role in the uptake and release of excitatory neurotransmitter glutamate [57]. When somehow the glutamate uptake is disturbed, excess glutamate leads to the release of neurotransmitters, synaptic transmission, and hyperexcitability in the surrounding neurons. Our study demonstrated a decrease of the EAAT1 protein after cis- or oxaliplatin exposure for 24 h. In an in vivo study in rats, spinal microdialysis revealed an elevated glutamate concentration in oxaliplatin-treated animals. Furthermore, glutamate transporter 1 (GLT-1) expression was decreased at the same time [58]. In paclitaxel-treated rats, the downregulation of both GLT-1 and EAAT1 has been observed in the dorsal horn. It has been discussed that the downregulation of glutamate transporters may contribute to paclitaxel-induced hyperalgesia [43]. In different studies of nerve injury models, the GLT-1 and the EAAT1 proteins were decreased. In one study, additionally, the location of the protein was altered. Under control conditions, only astrocytes expressed both the GLT-1 and the EAAT1 protein; however, after d 7 of the nerve injury, microglia started to express both proteins as well [59]. These data suggest a critical role for the EAAT1 protein in the induction and maintenance of painful neuropathies after chemotherapy.

Extracellular uptake of glutamate strongly depends on the hyperpolarized membrane potential. Our study demonstrates a direct link between the downregulation of K_ir4.1_ and EAAT1. Using a specific K_ir4.1_ blocker led to nearly completely blocked channel currents, resulting in reduced current densities and decreased resting membrane potentials. Furthermore, the specific block of the K_ir4.1_ led to a downregulation of the EAAT1 protein. Due to the changed membrane potential, the ability of the astrocytes to uptake glutamate was inhibited [53,60]. Depolarization of the astrocytic membrane by reduction or inhibition of the K_ir4.1_ channel leads to a reduced potassium uptake and increases the extracellular potassium concentration, with subsequent changes in the electrochemical potassium gradient. Glutamate uptake by EAAT proteins depends on the sodium influx and the potassium efflux. Due to the increase of extracellular potassium concentration by K_ir4.1_ dysfunction, potassium can no longer leave the cell through EAAT1, resulting in reduced glutamate uptake [61,62,63]. These results suggest the proper function of the K_ir4.1_ channel in order to reach the hyperpolarized membrane potential and favor the astrocytes glutamate uptake [64,65,66]. When cultured control astrocytes were exposed to K_ir4.1_ inhibitor VU, a reduction of EAAT1 expression was observed, supporting the relationship between these two proteins. Interestingly, this inhibition did not increase GFAP expression significantly, suggesting a K_ir4.1_ independent mechanism.

Minocycline is an antibiotic commonly used to treat bacterial infections. Several studies have demonstrated that it could act as an inhibitor for microglia and astrocytes in the spinal cord [67]. Minocycline can inhibit the spinal p38 mitogen-activated protein kinase (MAPK) and phosphoinositide 3-kinase (PI) signaling pathways [67,68]. It can also directly inhibit N-methyl-D-aspartate (NMDA) receptors or inhibit calcium influx [69]. This identifies minocycline as a potentially important mechanism in preventing neuropathic pain [68]. To demonstrate the direct activation of cis- or oxaliplatin on cultured astrocytes and potential neuroprotective properties, spinal astrocytes were exposed to chemotherapeutics and minocycline as well. Here, minocycline abolished all previously described cis- or oxaliplatin-induced effects. In oxaliplatin-treated rats, co-treatment with minocycline led to the reduction of activated astrocytes as well as oxaliplatin-induced allodynia [38]. Systemic treatment with minocycline in paclitaxel-treated rats prevented the activation of astrocytes and the downregulation of GLT-1 and EAAT1 in the spinal dorsal horn [43]. Oxaliplatin, paclitaxel, or bortezomib do not lead to activation of microglia (Zhang et al., 2012, Robinson et al., 2014), proving the protective effect of minocycline on astrocytes without the contribution of microglia, while Hu et al. described microglia activation in a mouse model of cisplatin-induced peripheral neuropathy [70]. However, we did not observe an increase of Iba-1 positive cells in our highly-enriched spinal astrocyte culture after exposure to cis- or oxaliplatin for 24 h. Nevertheless, microglia are a critical modulator of astrocytes’ function, suggesting a potential role in CIPN, at least mediated by cisplatin, which needs to be addressed in future studies. Our data demonstrate a direct influence of chemotherapeutics cis- or oxaliplatin on spinal astrocytes function and confirm the protective potential of minocycline in cis- or oxaliplatin-induced neuropathy. In particular, the reduction of glutamate uptake protein EAAT1 can be of broad interest to the field of CIPN. A reduction in glutamate uptake proteins on the spinal cord level can suggest a potential unbalance in the activation and inhibition of dorsal horn neurons. Further studies on the excitatory (glutamate) and inhibitory (GABA, glycine) transmitter systems are necessary to shed light on the contribution of these mechanisms to CIPN. Additionally, the influence of those drugs on neurons or glial cells of higher brain regions, such as the thalamus or hippocampus, needs to be evaluated in the future due to the knowledge of the involvement of these regions in painful sensations and their contribution to the phenomenon of “Chemo-brain”. This could be done with in vivo models of CIPN, different cell culture models, or organotypic slice cultures of these regions using the stated cis- or oxaliplatin concentrations.

## 4. Materials and Methods

### 4.1. Animals

Three- to four-week-old male and female Wistar rats (60–80 g, Animal Research Lab, University of Duisburg–Essen, Essen, Germany) were used to prepare cultures of spinal astrocytes. Experiments were performed according to the guidelines of the Animal Care and Use Committees of the University of Duisburg–Essen, Germany. All animals were kept on a 14-/10-h light/dark cycle with water and standard food pellets available ad libitum.

### 4.2. Isolation of Astrocytes from Spinal Cord

To isolate astrocytes from rat spinal cords, animals were deeply anesthetized by isoflurane, and the spinal column was dissected. The whole spinal cord was removed by hydraulic extrusion and, to avoid contamination of fibroblast, meninges covering the outer surface of the spinal cord were removed. Only the lumbar part of the spinal cord was used for astrocytes isolation. Spinal cord tissue was then chopped into a slurry using a razor blade and transferred to a 0.25% trypsin/EDTA solution for 30 min at 37 °C. Enzymatic digestion was stopped by adding DMEM/F12 + 10% FBS to the solution. Afterward, tissue was mechanically titrated until a cell suspension was formed.

The cell suspension was brought to 10 mL using DMEM/F12 containing 10% fetal bovine serum (FBS, Thermo Fisher Scientific, Dreiech, Germany) and 1% penicillin/streptomycin (PS, Thermo Fisher Scientific, Germany), placed into a 75-cm^2^ cell culture flask (T75), and incubated at 37 °C and 5% CO_2_. The next day, the medium was removed and replaced with fresh culture medium with 1 mM cytosine arabinoside (AraC). The medium was removed and replaced every two days with medium without AraC. After the cells reached approximately 65% confluency (10–14 days), to ensure a single-cell layer [71], flasks were shaken on an orbital shaker (200 rpm at 37 °C) overnight to remove microglia. Afterward, cells were trypsinized from the cell culture flask, counted, and placed on poly-d-lysine (PDL, Sigma-Aldrich, Taufkirchen, Germany)-treated glass coverslips in a 24-well plate (3500 cells per coverslip). The cells were cultured for 72 h and used in experiments.

The tissue of male and female rats was pooled and not checked for gender-specific differences.

### 4.3. Drug Application

Cisplatin (Abcam, Cambridge, UK) and oxaliplatin (Abcam, Cambridge, UK) were diluted with Aqua bidest at a concentration of 5 mM. Spinal astrocytes were exposed to 0.001 µM, 0.01 or 0.1 µM cisplatin or oxaliplatin for 24 h. This agrees with findings on oxaliplatin in the cerebrospinal fluid (CSF) of rats after a single intraperitoneal injection [34] and the findings for cisplatin in non-human primates [33], showing the relevance of the used concentrations for our hypothesis.

Antibiotic minocycline hydrochloride (minocycline, Sigma-Aldrich, Taufkirchen, Germany) was diluted in Aqua bidest and applied to spinal astrocytes in a concentration of 50 µg/mL 20 min before chemotherapeutics were applied.

Inward-rectifier potassium channel K_ir4.1_ inhibitor VU0134992 (VU, Tocris, Wiesbaden, Germany) was diluted with Aqua bidest, and cultured spinal astrocytes were exposed to 2 µM 20 min before application of chemotherapeutics.

### 4.4. Immunocytochemical Staining of Spinal Astrocytes

Spinal astrocytes cultures were fixed in 4% paraformaldehyde (PFA, Sigma-Aldrich, Taufkirchen, Germany) for 15 min and washed three times in phosphate-buffered saline (PBS). Cell membranes were permeabilized in PBS + 0.5% Triton X-100 for 15 min at room temperature. Nonspecific binding sites were blocked with 5% bovine serum albumin (BSA) in PBS for 1 h at room temperature.

To determine the purity of spinal astrocyte culture, the cells were incubated with primary antibodies specific for glial fibrillary acidic protein (anti-GFAP, rabbit, 1:400, Sigma-Aldrich, Taufkirchen, Germany), connexin-43 (anti-Cx-43, rabbit, 1:500; Sigma-Aldrich, Taufkirchen, Germany), S-100β (anti-S100β, rabbit, 1:200, Thermo Fisher Scientific, Dreieich, Germany), β-III-tubulin (anti-Tuj1, mouse, 1:500 Sigma-Aldrich, Taufkirchen, Germany), glutamine synthetase (anti-GS, rabbit; 1:400, Sigma-Aldrich, Taufkirchen, Germany), K_ir4.1_ (anti-KCNJ10, rabbit, 1:500, Alomone Labs, Jerusamlem, Israel), and Iba-1 (anti-Iba-1, rabbit, 1:500, Thermo Fisher Scientific, Dreiech, Germany).

The influence of cisplatin or oxaliplatin on protein levels was determined with GFAP (anti-GFAP, rabbit, 1:400; Sigma-Aldrich, Taufkirchen, Germany), EAAT1 (mouse, 1:500, Santa Cruz, Heidelberg, Germany), and K_ir4.1_ (anti-K_ir4.1_, rabbit, 1:500, Alomone Labs, Jerusalem, Israel).

After washing, spinal astrocytes were incubated with secondary antibodies (goat anti-mouse Cy3 or goat anti-rabbit Alexa Fluor 488, 1:500, Thermo Fisher Scientific, Dreieich, Germany) for 1 h in the dark at room temperature and counterstained with DAPI (1:500, Sigma-Aldrich, Taufkirchen, Germany) for DNA.

To determine the protein levels of GFAP, EAAT1, and K_ir4.1_, immunoreactivity was quantified using ImageJ software (NIH). Immunoreactive cells were selected using a freehand tool. Each protein’s integrated density in each immunoreactive positive spinal astrocyte was measured and normalized against each image’s background. The data were calculated from three to four independent replicates per protein.

### 4.5. Electrophysiology

To isolate I_Kir4.1_ from spinal astrocytes, whole-cell patch-clamp measurements were performed as described elsewhere [11,25,26]. Briefly, micropipettes were pulled from filament-containing borosilicate glass (1.5 mm OD × 0.86 mm ID × 100 mm L, Bio-Medical Instruments, Zöllnitz, Germany) with a pipette puller (Sutter Instruments, Novato, CA, USA) to have a resistance of 3–7 MΩ. The internal solution (pipette solution) consists of: 125 mM K-Gluconate, 2 mM CaCl_2_, 2 mM MgCl_2_, 10 mM EGTA, 10 HEPES, 0.5 mM Na-GTP, and 2 mM Na_2_ATP, pH with KOH (pH 7.2) and the external solution (bath solution) consists of: 14.4 mM NaHCO_3_, 5.9 mM KCl, 2. 5 mM MgSO_4_, 120.9 mM NaCl, 1.2 mM NaH_2_PO_4_, 2.5 mM CaCl_2_, and 11.5 mM glucose (pH 7.4). The patch-clamp measurement was performed at a defined voltage, and spinal astrocytes were clamped at −70 mV with a depolarization range from −180 mV to 40 mV (in increments of 20 mV) in order to monitor the current-voltage relation curve (IV) of I_Kir4.1_. The membrane potential, current density, and cell capacitance were additionally recorded.

Patch-clamp protocols were evaluated using the EPC10 amplifier, Patchmaster software (HEKA, Reutlingen, Germany) and Microsoft Excel. The Patchmaster software corrected “leakage currents” using an integrated P/4 protocol.

### 4.6. Statistical Analysis

For the statistical analysis of the data, Mann–Whitney–U or one-way ANOVA was used. Significances were defined at a value of *p* < 0.05. All values are means ± Standard Error of Mean (SEM).

## 5. Conclusions

In summary, we demonstrate the modulation and function of different astrocyte proteins linked to a direct impact of cis- or oxaliplatin on these cells. Furthermore, we observed K_ir4.1_ dependent downregulation of EAAT1 and an independent upregulation of GFAP as a sign of astrocytes activation. Due to this, spinal astrocytes may serve as a potential target, especially the K_ir4.1_ and the EAAT1 proteins, for prevention or therapeutic strategies.

## Data Availability

The data that support the findings of the study are available on request from the corresponding author.

## Supplementary Materials

Supplementary materials can be found at https://www.mdpi.com/article/10.3390/ijms22126300/s1.
