# Supplementary material for: Modulation of Glutamate Transporter EAAT1 and Inward-Rectifier Potassium Channel Kir4.1 Expression in Cultured Spinal Cord Astrocytes by Platinum-Based Chemotherapeutics
Source: Int J Mol Sci. 2021 Jun 11;22(12):6300. doi: 10.3390/ijms22126300 (PMC8230757; doi:10.3390/ijms22126300)
Supplement: Supplementary file 1 [file ijms-22-06300-s001.zip › ijms-1224023-supplementary.pdf]

Supplementary Material

Modulation of Glutamate Transporter EAAT1 and Inward-Rectifier Potassium Channel $K_{ir4.1}$ Expression in Cultured Spinal Cord Astrocytes by Platinum-Based Chemotherapeutics

Markus Leo *, Linda-Isabell Schmitt, Rebecca Steffen, Andrea Kutritz, Christoph Kleinschnitz and Tim Hagenacker

Department of Neurology , Center for Translational Neuro- and Behavioral Sciences (C-TNBS), University Hospital Essen, 45147 Essen, Germany; Linda-Isabell.Schmitt@UK-Essen.de (L.-I.S.); Rebecca.Steffen@stud.uni-due.de (R.S.); Andrea.Kutritz@UK-Essen.de (A.K.); Christoph.Kleinschnitz@UK-Essen.de (C.K.); Tim.Hagenacker@UK-Essen.de (T.H.)

* Correspondence: Markus.Leo@UK-Essen.de; Tel.: +49-201-723-82366

Methods

Immunocytochemical staining of spinal astrocytes

Spinal astrocytes cultures were fixed in 4 % paraformaldehyde (PFA, Sigma Aldrich, Germany) for 15 min and washed three times in phosphate-buffered saline (PBS). Cell membranes were permeabilized in PBS + 0.5 % Triton X-100 for 15 min at room temperature. Nonspecific binding sites were blocked with 5 % bovine serum albumin (BSA) in PBS for 1 h at room temperature.

For determining the activation of spinal astrocytes by cis- or oxaliplatin, astrocytes were incubated with primary antibodies specific for S-100β (anti-S100β, rabbit, 1:200, Thermo Fisher Scientific, Germany).

For determining the number of Iba-1 positive cells in cultured spinal astrocytes, cells were incubated with primary antibody specific for EAAT1 (anti-EAAT1, guinea pig, 1:500, Synaptic Systems, Germany) and Iba-1 (anti-Iba-1, rabbit, 1:500, Thermo Fisher Scientific, Germany).

After washing, spinal astrocytes were incubated with secondary antibodies (goat anti-guinea pig Cy3 or goat anti-rabbit Alexa Fluor 488, 1:500, Thermo Fisher Scientific, Germany) for 1 h in the dark at room temperature and counterstained with DAPI (1:500, Sigma Aldrich, Germany) for DNA.

To determine the protein levels of S100β, immunoreactivity was quantified using ImageJ software (NIH). Immunoreactive cells were selected using a freehand tool. Each protein's integrated density in each immunoreactive positive spinal astrocyte was measured and normalized against each image's background. The data were calculated from three to four independent replicates per protein.

Production of reactive oxygen species (ROS)

According to the manufacturer's protocol, to measure the ROS production in cultured spinal astrocytes after exposure to cis- or oxaliplatin for 24 h, CellRox Assay (Thermo Fisher Scientific, Germany) was performed.

Images were obtained using a Zeiss Axio Examiner fluorescence microscope, and the fluorescence intensity was analyzed using ImageJ software (NIH). After cisplatin exposure, the ROS intensity was normalized to the intensity of the untreated spinal astrocytes.

Results

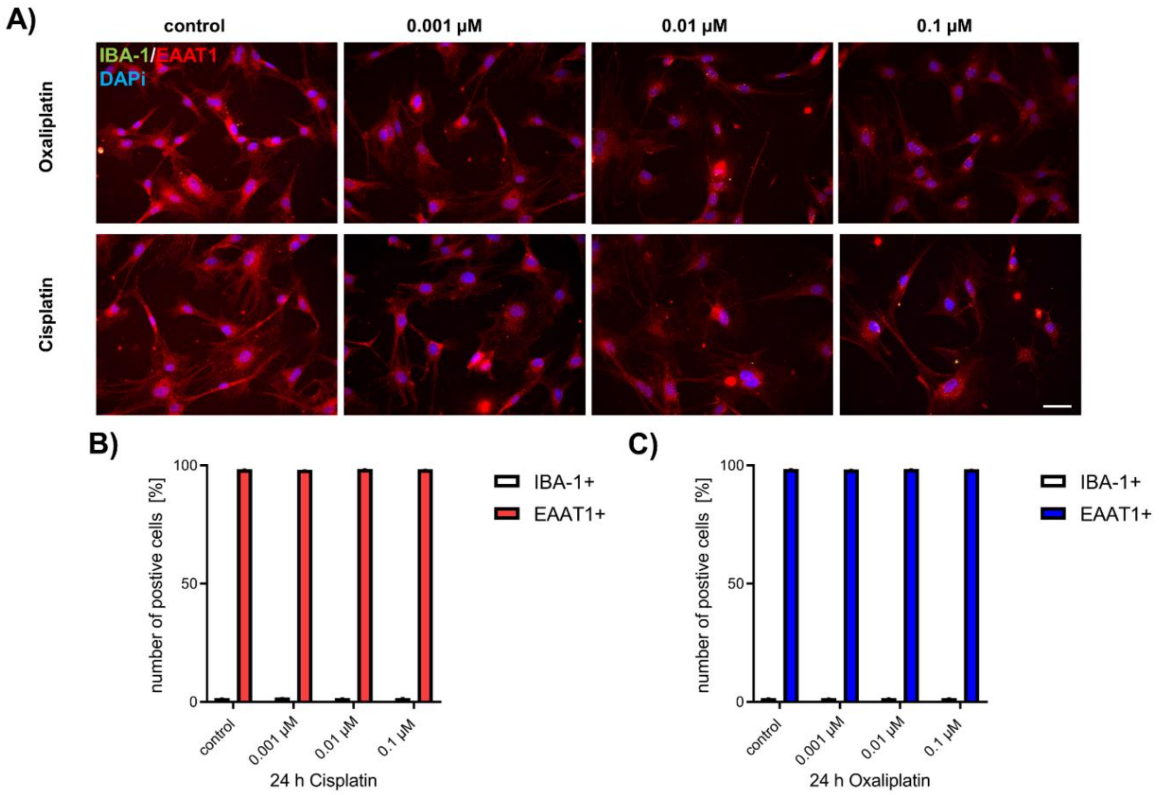

Figure S1. Number of Iba-1 positive cells cultured spinal astrocytes before and after exposure to cis- or oxaliplatin. (**A**) Immunocytochemical staining of EAAT1 (red) as a marker of astrocytes and Iba-1 (green) as a marker of non-astrocyte cells as microglia in cultured spinal astrocytes before and after exposure to 0.001 µM, 0.01 µM, or 0.1 µM cis- or oxaliplatin for 24 h. Nuclear DNA was stained with Dapi (blue). (**B**) Exposure of spinal astrocytes to 0.001 µM cisplatin did not affect the number of Iba-1 positive cells (p>0.05). n = 4 individual experiments with >100 cells. Scale 50 µm.

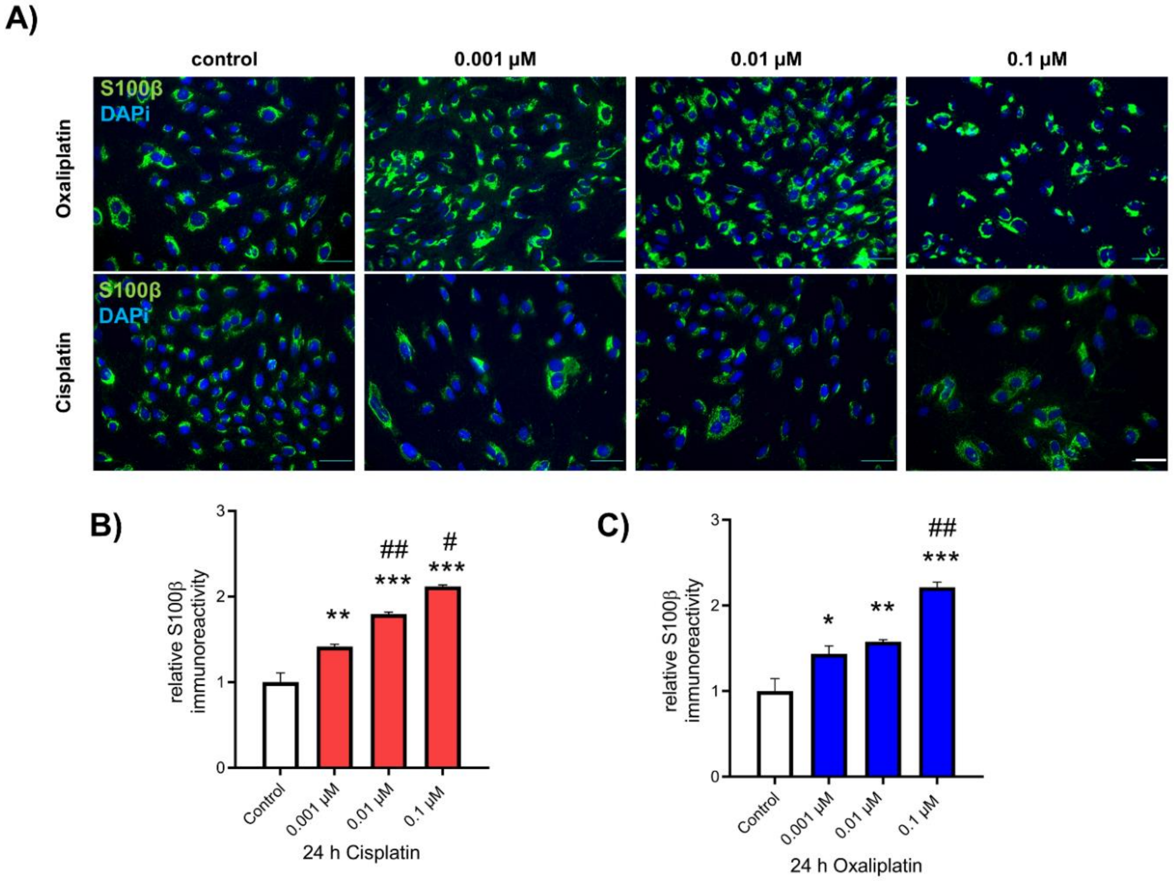

Figure S2. Expression of S100β in cultured spinal astrocytes before and after exposure to cis- or oxaliplatin. (**A**) Immunocytochemical staining of S100β (green) in cultured spinal astrocytes before and after exposure to 0.001 µM, 0.01 µM, or 0.1 µM cis- or oxaliplatin for 24 h. Nuclear DNA was stained with Dapi (blue). (**B**) Exposure of spinal astrocytes to all concentrations of cisplatin for 24 h led to an increase of S100β expression (p<0.01, ** - p<0.001, ***). A concentration-dependent effect was observed (p<0.05, # - p<0.01, ##). (**C**) Exposure of spinal astrocytes to all concentrations of oxaliplatin for 24 h led increase in S100β expression (p<0.05, * - p<0.001; ***). A concentration-dependent effect between 0.01 µM and 0.1 µM was observed (p<0.01; ##). * = significant difference to control. # = significant difference to previous concentration. n = 9 individual experiments with >100 cells. Scale 50 µm.

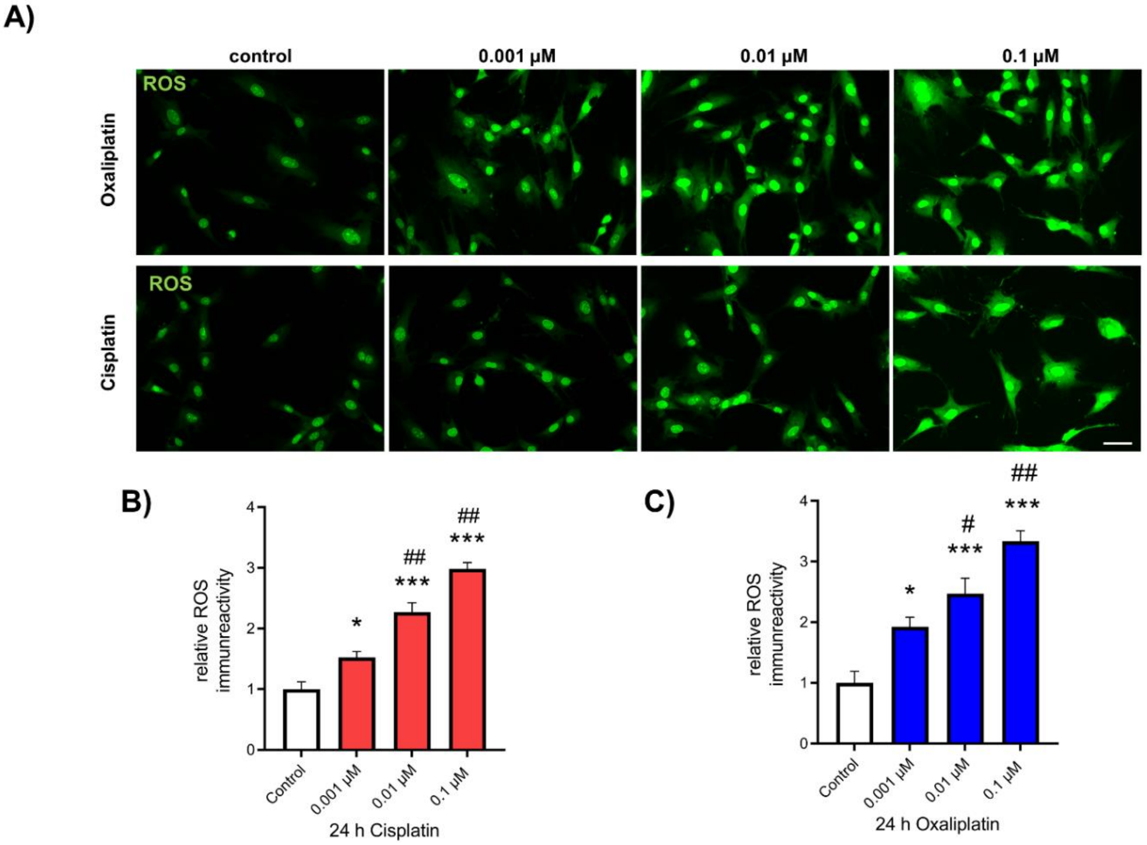

Figure S3. Production of ROS in cultured spinal astrocytes before and after exposure to cis- or oxaliplatin. (**A**) Immunocytochemical staining of ROS (green) in cultured spinal astrocytes before and after exposure to 0.001 µM, 0.01 µM, or 0.1 µM cis- or oxaliplatin for 24 h. (**B**) Exposure of spinal astrocytes to all concentrations of cisplatin for 24 h increased the production of ROS (p<0.05, * – p<0.001, ***). A concentration-dependent effect was shown (p<0.01; ##). (**C**) Exposure of spinal astrocytes to all concentrations of oxaliplatin for 24 h led to an increase of ROS production (p<0.05, *** - p<0.001; ***). A concentration-dependent effect was observed (p<0.05, # - p<0.01, ##). * = significant difference to control. # = significant difference to previous concentration. n = 4 individual experiments with >100 cells. Scale 50 µm.
